# Java Ranger at SV-COMP 2020 (Competition Contribution)

**DOI:** 10.1007/978-3-030-45237-7_27

**Published:** 2020-03-13

**Authors:** Vaibhav Sharma, Soha Hussein, Michael W. Whalen, Stephen McCamant, Willem Visser

**Affiliations:** 8 Johannes Kepler University, Linz, Austria; 9 University of Birmingham, Birmingham, UK; 10 University of Minnesota, Minneapolis, MN USA; 11 Ain Shams University, Cairo, Egypt; 12 Stellenbosch University, Stellenbosch, South Africa

## Abstract

Path-merging is a known technique for accelerating symbolic execution. One technique, named “veritesting” by Avgerinos et al. uses summaries of bounded control-flow regions and has been shown to accelerate symbolic execution of binary code. But, when applied to symbolic execution of Java code, veritesting needs to be extended to summarize dynamically dispatched methods and exceptional control-flow. Such an extension of veritesting has been implemented in Java Ranger by implementing as an extension of Symbolic PathFinder, a symbolic executor for Java bytecode. In this paper, we briefly describe the architecture of Java Ranger and describe its setup for SV-COMP 2020.

## 1 Approach

Symbolic execution is a well-known program analysis technique that has been applied to many applications such as test generation [3,7], equivalence checking [6,8], and vulnerability finding [13]. However, when applied to large software, symbolic execution can suffer from scalability challenges caused by path explosion. Path-merging techniques such as veritesting [1] and dynamic state merging [4] help alleviate these scalability limitations. In particular, veritesting attempts to construct a static summary of a multi-path region and use it. Veritesting has been shown to significantly accelerate symbolic execution of binary code. Given that a large amount of software in use today is still written in Java, it is desirable to bring the benefits of veritesting to symbolic execution of Java as well. However, features such as dynamic dispatch make path-merging for Java code challenging [11]. The summary of a multi-path region that contains a dynamically-dispatched method call can only be constructed if the method to be called can also be summarized. Java Ranger (JR) extends the current state-of-the-art path-merging ideas presented by Avgerinos et al. [1] by first building static summaries which are later transformed using runtime information such as

* Jury Member the dynamic type of an object reference used for accessing a field. Java Ranger is built as an extension to Symbolic PathFinder (SPF) [5].

## 2 Architecture

Java Ranger is implemented as an SPF listener that watches for symbolic branch conditions in branching instructions. On encountering a symbolic branch instruction, JR attempts to create a summary for the multi-path region that begins at that branch instruction and ends at its exit points. A multi-path region is a region of code that begins at a branch instruction with a symbolic branch condition. An exit point of a multi-path region is either (1) the first program location in a control-flow path through the multi-path region which could not be summarized, or (2) the location of the immediate post-dominator of the multi-path region. This mechanism is also explained by Sharma et al. [12] in Figure 4.

## 3 Strengths And Weaknesses

Since JR improves scalability limitations of symbolic execution, its strength can only be observed when running it over large software. However, JR falls back to vanilla symbolic execution when it finds no opportunity for path-merging. SV-COMP 2020 had 416 verification tasks in the Java track. More information on SV-COMP 2020 can be found in its competition report [2]. JR instantiated at least one static summary on 96 different benchmarks of the 416 benchmarks. The summary for a multi-path region can be instantiated more than once on each benchmark because it is possible that the symbolic executor will encounter the same multi-path region more than once while running the benchmark. In total, JR instantiated 356 unique summaries. The total number of instantiated summaries used by JR was 20,182. JR also inlined a method summary a total of 62,857 times while instantiating these summaries.

JR also had a "unknown" conclusion on 40 of the 416 SV-COMP 2020 verification tasks. 22 of the 40 were caused due to our JR configuration which turned off support for symbolic strings because we found SPF's support for solving string constraints was not stable. 9 "unknown" conclusions were reached due to missing support for symbolic array lengths in multi-dimensional arrays. 8 of the 40 occurred due to a timeout. The last "unknown" result occurs in the equivalence check verification task in the ApacheCLI benchmark due to JR's use of a depth limit.

We made use of two depth limit parameters in SV-COMP 2020. The first was a limit on the exploration depth of our baseline symbolic executor, SPF. The second was a depth limit on the recursive depth to which our method summaries would be inlined. While we wished to avoid the use of any such limit, we found similar kinds of limits were used by many participanting tools in SV-COMP 2019. It is common to use some kind of limitation when applying symbolic execution tools in practice, since they can get bogged down by path explosion or related problems, and path-merging helps with but does not eliminate this issue.

The Java verification category of SV-COMP 2020 did not score a tool's answer differently if it used a depth limit for producing that answer. Instead, the use of depth limit is reflected in each tool's score only if it caused the tool to produce an incorrect answer. We describe these depth limits and JR's configuration options in the following section.

## 4 Tool Setup and Configuration

Java Ranger's setup is very similar to the setup used by SPF. Since Java Ranger is simply an extension of SPF, the Java Ranger directory can be specified as a valid jpf-symbc extension of JPF. A JR configuration requires the following additions.

veritestingMode = <1-5>

veritestingMode specifies the path-merging features to be enabled with each higher number adding a new feature to the set of features enabled by the previous number. Setting veritestingMode to 1 runs vanilla SPF. Setting it to 2 enables path-merging for multi-path regions with no method calls and a single exit point. Setting it to 3 adds path-merging for multi-path regions that make method calls where the method can be summarized by Java Ranger. Setting it to 4 adds path-merging for multi-path regions with more than one exit point caused due to exceptional behavior and unsummarized method calls. Setting it to 5 adds path-merging for summarizing return instructions in multi-path regions by treating them as an additional exit point.

performanceMode = <true or false>

Setting performanceMode to true causes Java Ranger to minimize the number of solver calls to check the feasibility of the path condition when summarizing a multi-path region with multiple exit points.

TARGET_CLASSPATH_WALA=<classpath of target code>

Java Ranger needs this variable to be set up as environment variable. It is not part of the .jpf configuration file. This environment variable tells Java Ranger where it should be expecting to find code that needs to be statically summarized.

jitAnalysis=<true or false>

When turned on (the default value), this option causes JR to summarize multi-path regions when it encounters them. When turned off, JR attempts to summarize all multi-path regions reachable in a statically-computed interprocedural call graph up to a configurable limit.

recursiveDepth=<an integer value>

This option forces JR to restrict inlining of method summaries up to the value provided for this option. We set this parameter to 12 for SV-COMP 2020.

The following option is a JPF [14] configuration option which we also used for SV-COMP 2020.

search.depth_limit=<an integer value>

This option forces JPF to restrict its exploration to the depth provided as the value for this option. JPF constructs a tree of possible choices and explores the tree in a heuristic order, depth-first by default. Since JR is built as an extension to SPF, which is in turn built as an extension to JPF, we were able to restrict JR's exploration of choices using this option. We set this parameter to the value 13 for SV-COMP 2020.

## 5 Software Project and Contributors

Java Ranger is an extension of SPF. It is maintained on GitHub [9]. The version of Java Ranger that participated in Sv-COMP 2020 is publicly available [10]. For more information, please contact the authors of this paper.

## 6 Acknowledgments

The research described in this paper has been supported in part by the National Science Foundation under grant 1563920.
